# MFA-net: Object detection for complex X-ray cargo and baggage security imagery

**DOI:** 10.1371/journal.pone.0272961

**Published:** 2022-09-01

**Authors:** Thanaporn Viriyasaranon, Seung-Hoon Chae, Jang-Hwan Choi

**Affiliations:** 1 Division of Mechanical and Biomedical Engineering, Graduate Program in System Health Science and Engineering, Ewha Womans University, Seoul, South Korea; 2 Welfare and Medical ICT Research Department, Electronics and Telecommunications Research Institute, Daejeon, South Korea; University of Engineering & Technology, Taxila, PAKISTAN

## Abstract

Deep convolutional networks have been developed to detect prohibited items for automated inspection of X-ray screening systems in the transport security system. To our knowledge, the existing frameworks were developed to recognize threats using only baggage security X-ray scans. Therefore, the detection accuracy in other domains of security X-ray scans, such as cargo X-ray scans, cannot be ensured. We propose an object detection method for efficiently detecting contraband items in both cargo and baggage for X-ray security scans. The proposed network, MFA-net, consists of three plug-and-play modules, including the multiscale dilated convolutional module, fusion feature pyramid network, and auxiliary point detection head. First, the multiscale dilated convolutional module converts the standard convolution of the detector backbone to a conditional convolution by aggregating the features from multiple dilated convolutions using dynamic feature selection to overcome the object-scale variant issue. Second, the fusion feature pyramid network combines the proposed attention and fusion modules to enhance multiscale object recognition and alleviate the object and occlusion problem. Third, the auxiliary point detection head adopts an auxiliary head to predict the new keypoints of the bounding box to emphasize the localizability without requiring further ground-truth information. We tested the performance of the MFA-net on two large-scale X-ray security image datasets from different domains: a Security Inspection X-ray (SIXray) dataset in the baggage domain and our dataset, named CargoX, in the cargo domain. Moreover, MFA-net outperformed state-of-the-art object detectors in both domains. Thus, adopting the proposed modules can further increase the detection capability of the current object detectors on X-ray security images.

## Introduction

X-ray screening systems support transport security by detecting concealed prohibited items, including explosives, drugs, weapons, chemicals, and other contraband in carry-on baggage and cargo. In traditional X-ray screening systems, manual screening by human operators plays a vital role. However, complex features in X-ray images, such as object occlusion, and object-scale variety, increase in the fatigue level of the screener, which could cause false alarms. Therefore, automatic detection of prohibited items, especially employing artificial intelligence (AI)-based X-ray scanning, is deployed to decrease the processing time and increase the accuracy of the detection process.

**Fig 1:**
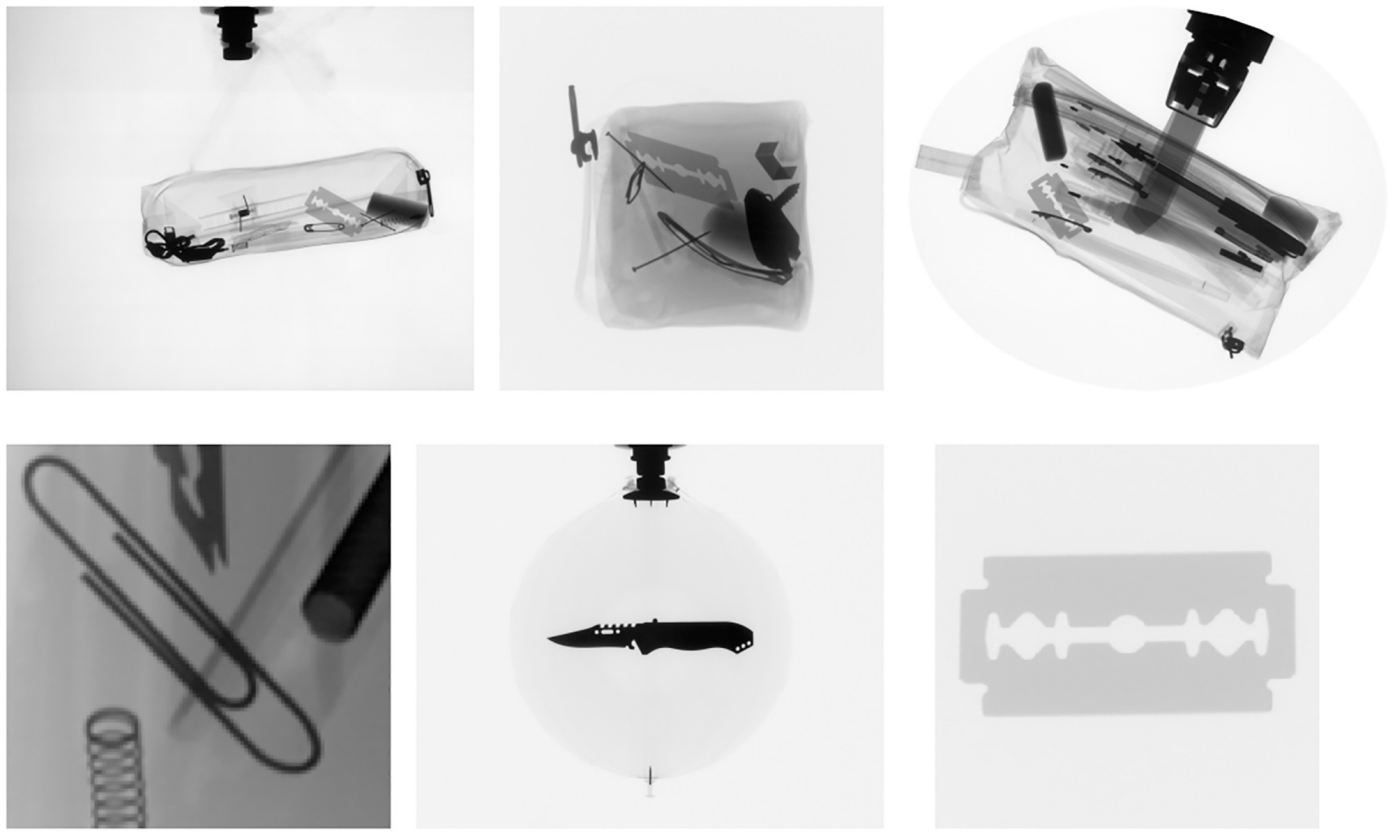
Representative image examples of the GDXray dataset [1].

A critical challenge of AI-based X-ray inspection system development is the limited availability of X-ray security image datasets of good quality and large size. Only a few X-ray screening image dataset are publicly available. For example, GDXray [1] is a small publicly released benchmark dataset of baggage X-ray scanning images with noncomplex content, as presented in Fig 1. In addition, SIXray [2] is a large benchmark dataset of baggage X-ray scanning images that provides more challenging detection images than GDXray in terms of complex content anFd bias-negative samples (*i.e.*, images without prohibited items). To our knowledge, public benchmark datasets of X-ray security images only contain images from the baggage domain. However, X-ray scanning systems are used in baggage scanning and other environments, such as cargo scanning. Therefore, AI-based systems trained using these public datasets [3–7] might be inadequate for dealing with real-world scenarios. In this work, we first synthesized a cargo X-ray scanning image dataset called CargoX, which provides complex content that includes variation geometrics with varying scales and viewpoints of objects. Furthermore, CargoX provides further information for detection learning (*i.e.*, instance segmentation information). Accordingly, we evaluated the performance of existing AI-based object detectors and the proposed object detector on the SIXray and CargoX datasets to explore the compatibility of detectors in real-world security X-ray scanning scenarios.

Recently, AI-based security scanning for classification and prohibited object detection has had rapid advances with the use of traditional machine learning [8–13] and deep convolution neural networks (CNNs) [2, 14–21]. Previous studies on objection detection within the context of baggage threat recognition have employed state-of-the-art object detectors with transfer learning to overcome the limitations of the small sizes X-ray security datasets. However, although transfer learning can enhance detection performance, it is still sensitive to geometric variations, one of the main challenges in X-ray security images. Conditional convolution, such as dynamic convolution [22], SKNet [23], ResNeSt [24], switchable atrous convolution (SAC) [25], and deformable convolution [26], is an effective method to alleviate geometric variations in the natural image domain. Due to the heavy object-scale variant on X-ray security compared to the natural image domian, adding the existing condition convolutional method cannot achieve as high performance as the natural image domain as presented in Table 2. To increase the performance of the object detectors on X-ray security images, we propose using a new conditional convolution-based module called the multiscale dilated convolutional (MDConv) module. The MDConv module converts the standard convolution to a conditional convolution to overcome heavy geometric variations of prohibited items in the X-ray security dataset using multiple options for the dilated convolution. This method improves the performance of the geometric transformation of the detectors and fuses the features dynamically using the attention mechanism weight selection. As presented in Table 2, the MDConv module outperforms previous conditional convolutional methods and achieves mean average precision (mAP) values of 55.4% and 72.5% on the SIXray10 and CargoX datasets, respectively.

Another effective approach to addressing geometric variations, especially scale variations, is adopting the multiscale feature fusion network. The feature pyramid network (FPN) [27] is a representative multiscale feature fusion framework that achieves remarkable performance in object detection by fusing multiscale features with shallow content-descriptive and deep semantic features. After the success of the FPN, several FPN-based methods, such as the PAFPN [28], have been proposed to improve FPN performance. Cross-scale fusion and skip connections are general approaches to improve feature representation and alleviating information loss of the FPN (*e.g.*, Libra R-CNN [29], and AugFPN [30]). However, the fusion of differences in cross-scale feature maps after interpolation may cause aliasing effects, resulting in confused localization. As the attention mechanism can optimize the fused aliasing feature and enhance discriminative abilities [31], we propose an attention mechanism with multiscale feature fusion modules to create the feature fusion network (FusionFPN) to mitigate the information loss and aliasing effects and effectively generate multiscale semantic features to overcome object-scale variations and occlusion issues. As observed in Table 3, the FusionFPN outperforms the previous FPN-based methods and achieves mAP values of 54.6% and 71.3% on the SIXray10 and CargoX datasets, respectively.

Generally, bounding box regression is designed to predict the bounding box position defined by four points (*i.e.*, the width, height, and top-left corner *x-* and *y-* coordinates), which is enough for the localization of the object. However, bounding box prediction is not precise, especially on X-ray security images with complex content and heavy occlusion. We integrate the proposed the additional auxiliary head approach with the two-stage object detector detection head to achieve high localizability in X-ray security images. The proposed method is called the auxiliary point detection head (point head). The point head is designed to predict keypoints of the object bounding box to improve the localization performance of the detectors and mitigate the occlusion issue by precisely predicting the object bounding box. As presented in Table 5, the point head outperforms the previous detection head optimization method with an mAP value of 71.5% on the CargoX dataset.

Finally, we propose MFA-net, in which the MDConv module, FusionFPN, and point head described above are incorporated into a two-stage object detector to overcome the difficulties in X-ray security object detection systems, such as object-scale variance and object occlusion and enhance the localization performance of the detector on X-ray scanning images in baggage and cargo image domains. To demonstrate the effectiveness of the MFA-net, we incorporate the MFA-net into the Cascade R-CNN and Cascade Mask R-CNN and evaluate the detector performance on the SIXray and the synthetic dataset, CargoX. On the CargoX dataset, the MFA-net-based Cascade Mask R-CNN achieves an mAP value of 75.2%, 2.6% higher than that of the baseline Cascade Mask R-CNN. In addition, the mAP of the MFA-net-based Cascade R-CNN is 5.5% higher than the baseline Cascade R-CNN with an mAP of 58.0% on the SIXray10 dataset.

## Materials and methods

### MFA-net

**Fig 2:**
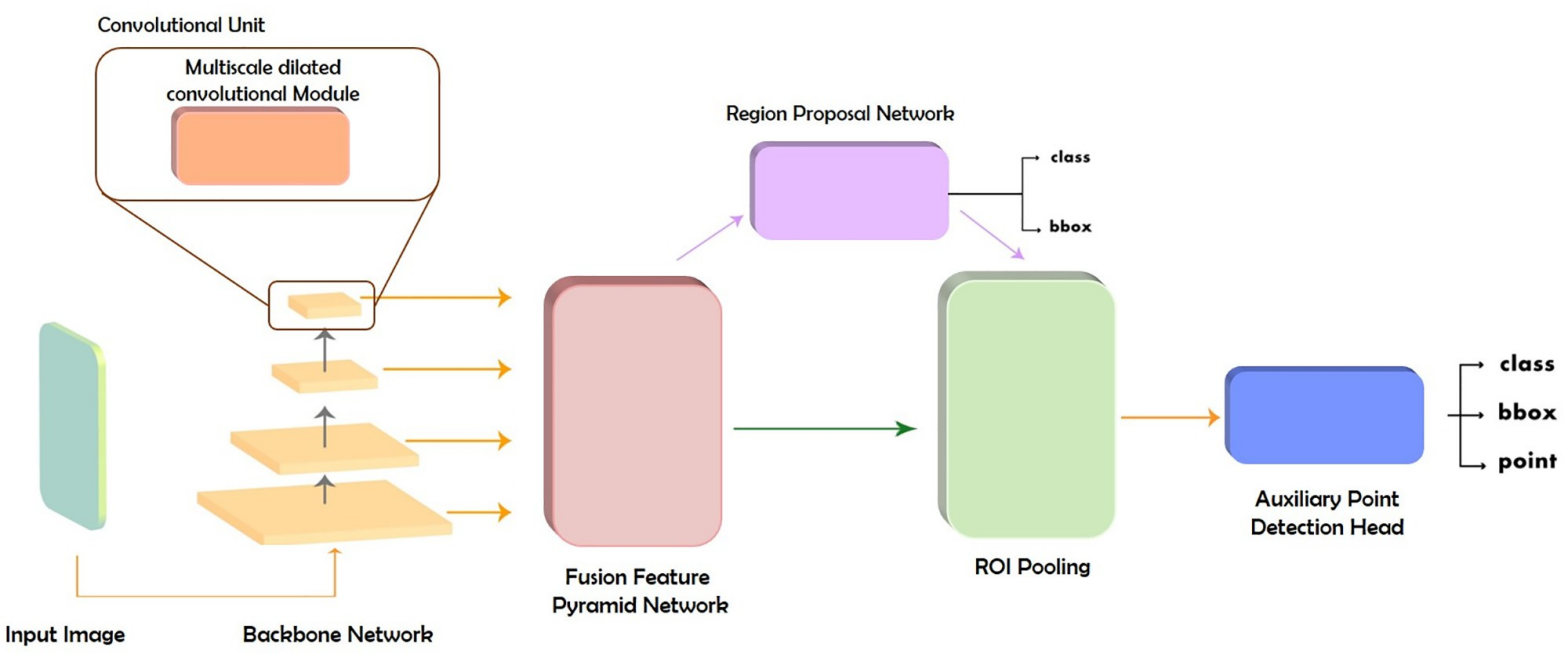
Two-stage object detector incorporated with the three proposed components (*i.e.*, the multiscale dilated convolutional module, fusion feature pyramid network, and auxiliary point detection head).

In this subsection, we describe the architectural design of the proposed MFA-net, which combines the two-stage object detectors with the three proposed components. The three proposed components (*i.e.*, MDConv module, FusionFPN, and point head) are designed to be incorporated into two-stage object detectors (*e.g.*, the Faster R-CNN [32], Cascade R-CNN, and Cascade Mask R-CNN [33]) on the backbone network, feature fusion network, and detection head, respectively, as depicted in Fig 2. Typically, the three proposed components can be incorporated into one- or two-stage object detectors. Nonetheless, the MFA-net aims to outperform state-of-the-art object detectors in terms of detection accuracy. Consequently, we incorporated the proposed methods with two-stage object detectors, demonstrating better detection accuracy than one-stage object detection. The architecture and details for each component are described in the following subsections.

### Multiscale dilated convolutional module

Generally, the backbone of state-of-the-art object detectors is the network used for the classification task (*e.g.,*, ResNet [34], VGG16 [35], and ResNeXt [36]) without the last fully connected layers. However, directly using the classification network as a backbone impedes the localization accuracy for large objects and the recognition of small objects. Therefore, we exploited dilated convolution, an effective technique to enlarge the field of view of filters or the kernel of convolution on the MDConv module to increase the multiscale object feature extraction ability of the detector backbone.

The MDConv module is designed to convert the 3×3 standard convolutional mechanism in all bottleneck blocks of the ResNet family classification backbone (*i.e.*, ResNet50 [34], ResNet101, and ResNeXt101 [36]) to 3×3 conditional convolution using a set of *k* parallel convolutional operations with diverse dilated rates and attention mechanism as the feature selection weight generator. The 3 ×3 standard convolutional operation with weight *W* and dilated rate *d* that takes *x* as input to generate an output *y*, where *y* = *Conv*(*x*, *W*, *d*), is converted to the conditional convolutional operation as follows:

$$\begin{array}{c} y=\sum_{i=1}^{k}\alpha_{i}Conv\left(x,W,d_{i}\right), \end{array}$$ (1)

where *k* is the number of parallel convolutional operations, *d*_*i*_ represents the dilated rate of each convolutional operation, and *α*_*i*_ denotes the feature selection weight for each convolutional operation generated by the selection module. In this study, we set *k* to 3 and *d*_*i*_ to 1, 2, and 4.

**Fig 3:**
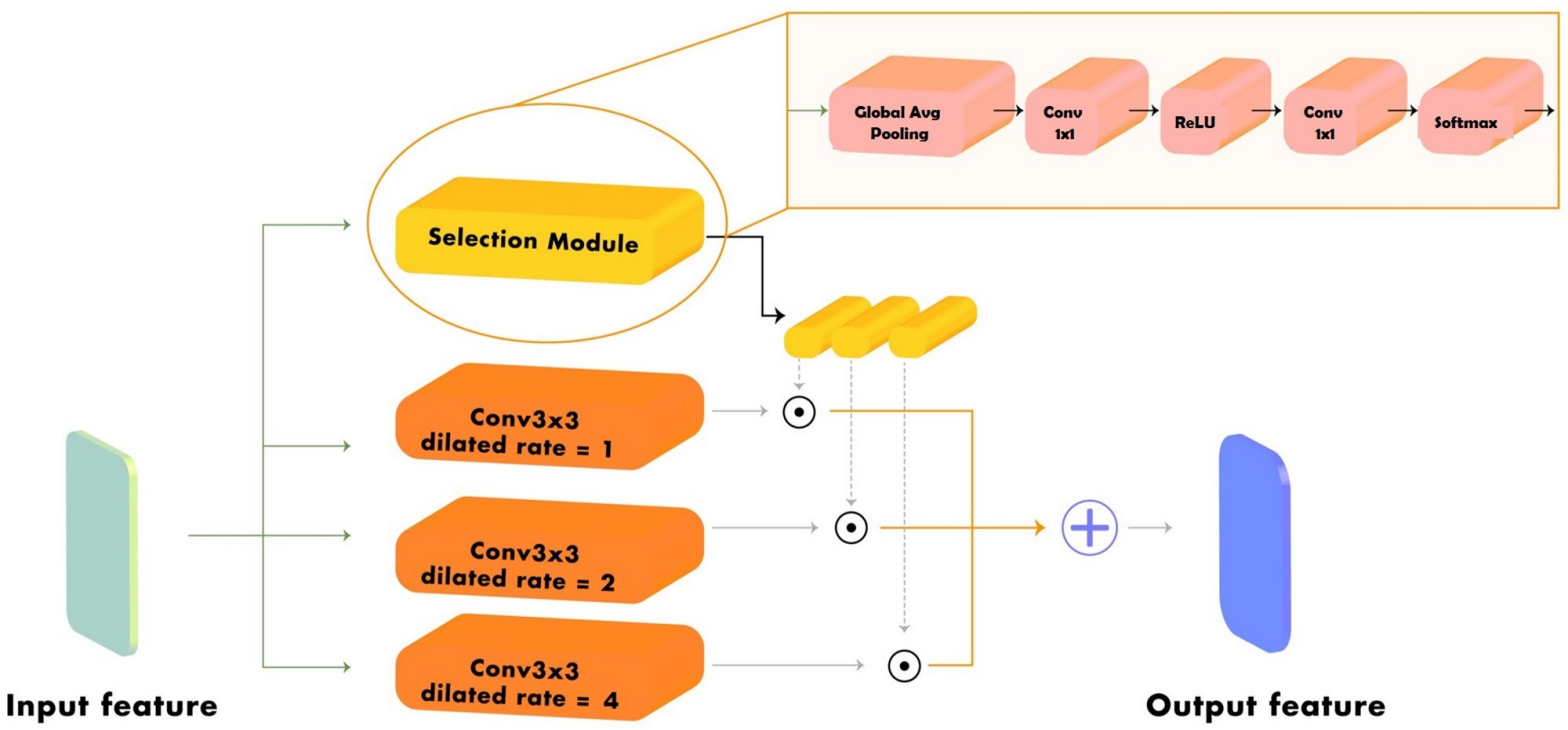
Multiscale dilated convolutional module architecture.

In addition, Fig 3 presents the architecture of the MDConv module, comprising three major processes. First, MDConv extracts the semantic feature from the output feature of the previous layers in the same scenarios as the input of the bottleneck layers of ResNet family backbone with *k* parallel 3×3 convolutional operations with various dilated rates. Second, the selection module generates the MDConv module input feature-dependent selection weight, *α*, for each individual output feature of the multiple-dilated-rates convolutional operation in the previous process. The feature selection weight is calibrated with the extracted global context. The architecture of the selection module consists of the global average pooling operation, 1×1 convolutional layers, rectified linear unit (ReLU) activation function and softmax activation function to generate and normalize the feature selection weight as follow.

$$\begin{array}{c} \alpha =\sigma (Conv(ReLU(Conv(GAP(x))))), \end{array}$$ (2)

where *x* is the input of the MDConv module, *GAP* denotes the global average pooling layer, *Conv* is a 1×1 convolutional layer, *ReLU* represents the ReLU activation function, and *σ* indicates a softmax activation function. Finally, the extracted features from the parallel convolutional operation are dynamically aggregated with the input feature-dependent selection weight with a multiplication operation that emphasize the most prohibited item-related features extracted from each 3×3 convolution operation in MDconv module and suppresses unrelated features.

### Fusion feature pyramid network

**Fig 4:**
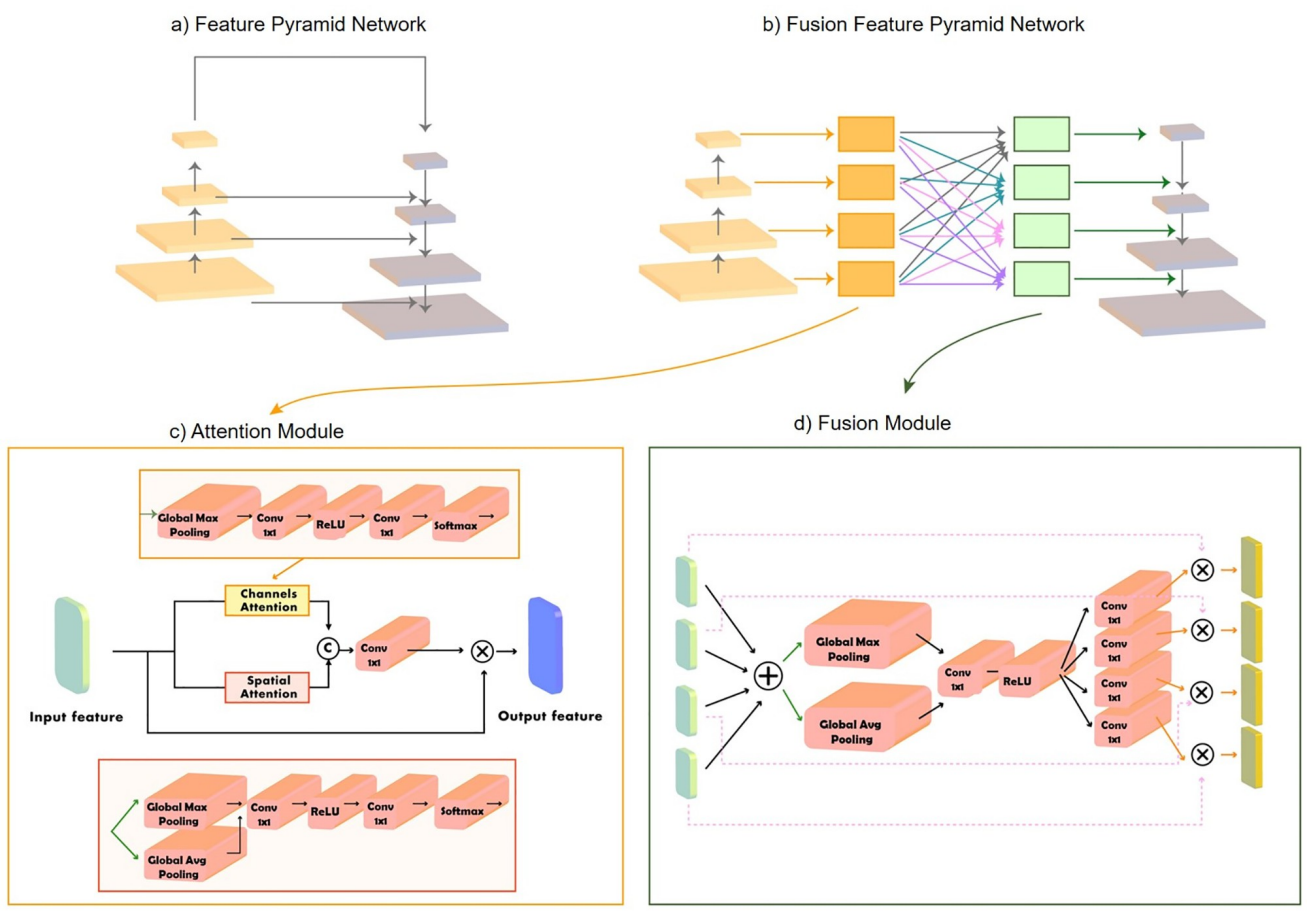
Comparison of the architecture of the feature pyramid network (FPN) and the proposed FusionFPN: a) architecture of the FPN, b) architecture of FusionFPN, c) attention module architecture, and d) fusion module architecture.

In general, a two-stage object detector backbone is equipped with a feature fusion network, such as the FPN [27], to achieve high performance by exploiting the inherent multiscale structure of the backbone to construct a feature pyramid with rich semantics at all levels, facilitating the detection of objects at different scales, as displayed in Fig 4a). However, the features at different levels in the FPN and FPN-based methods undergo a 1×1 convolution before performing feature fusion in a top-down path, leading to feature information loss at the highest pyramid level. Although cross-scale fusion and skip connections in FPN-based methods [29, 30] can alleviate the information loss, these methods result in an aliasing effect on integrated features. Consequently, we propose the FusionFPN to improve the localization performance of the detectors by mitigating the information loss of the FPN and the aliasing effect from different scale feature integrations. The FusionFPN further leverages the information from different levels of the feature pyramid by extending the FPN with an attention module and feature fusion module.

The FusionFPN has two major components: the attention and fusion modules incorporated sequentially (Fig 4b)). The attention module emphasizes the prohibited object-related features before the feature fusion process. Furthermore, the fusion module aggregates all scale features from the attention module to overcome the information loss problem of the original FPN. To mitigate the aliasing effect (*i.e.*, the influence of all scale-feature integration), we employ an attention mechanism, which is the intuitive solution to reduce the influence of the aliasing effects in the fusion module. In other words, the attention module is designed to improve the feature representation, and the fusion module is designed to alleviate the information loss of the original FPN with cross-scale feature fusion and reduce the aliasing effect of cross-scale feature fusion to improve the localizability of the model.

#### Attention module

The proposed attention module computes complementary attention in spatial and channel dimensions by incorporating channel attention and spatial attention in parallel. The attention maps from the two branches are fused via concatenation and undergo a 1×1 convolutional layer, as illustrated in Fig 4c). The channel attention module generates attention maps using the global context module. The module adopts global average pooling layers, effectively enhancing the learning of the extent of the object, followed by a 1×1 convolutional layer with the ReLU activation function and a 1×1 convolutional layer. In addition, the softmax activation function is employed in the last process as follows:

$$\begin{array}{c} A_{c}=\sigma \left(Conv\left(ReLU\left(Conv\left(GAP\left(F\right)\right)\right)\right)\right), \end{array}$$ (3)

where *F* is an intermediate feature map defined as the input of the attention module, *GAP* denotes the global average pooling layer, *Conv* is a 1×1 convolutional layer, *ReLU* represents the ReLU activation function, *A*_*c*_ indicates the channel attention map, and *σ* is a softmax activation function.

We employed global average pooling and global max pooling operations along the channel axis and aggregated them to generate an efficient feature descriptor to compute the spatial attention map. Global average pooling and global max pooling are applied to effectively highlight information regions and aggregate the information. The spatial information feature maps were forwarded to the 1×1 convolutional layer with the ReLU activation function and the 1×1 convolutional layer with a softmax activation function, as follows.

$$\begin{array}{c} A_{s}=\sigma \left(Conv\left(ReLU\left(Conv\left(GAP\left(F\right)+MAP\left(F\right)\right)\right)\right)\right), \end{array}$$ (4)

where *MAP* indicates the global max pooling layer, and *A*_*s*_ denotes the spatial attention map. The spatial attention map and channels attention were fused via concatenation, followed by a 1×1 convolutional layer. The fused attention map is multiplied by the input feature map using the elementwise multiplication operation to emphasize the prohibited object related feature in the input feature map. The overall attention process can be summarized as follows:

$$\begin{array}{c} F^{\prime }=Conv\left(concat\left(A_{c},A_{s}\right)\right)⊗F, \end{array}$$ (5)

where *F*′ is the output of the attention module, and *concat* is the concatenation process along the channel axis.

#### Fusion module

The proposed fusion module is based on a dynamic selection mechanism that allows the model to adaptively select the feature map from all levels of output of the attention module based on multiple levels of input information. The proposed fusion module consists of the fuse and select operations, as illustrated in Fig 4d). The fuse operation combines and aggregates the information from multiple levels to obtain global and comprehensive feature representation. The select operation aggregates the feature maps of differently levels according to the selection weights.

First, we prepared the input of the fusion module by resizing the different scale feature maps from attention module to the same spatial and channel dimension. We converted the channel dimension of the feature maps with a 1×1 convolutional operation (*Conv*). To resize the spatial feature map, we upsampled it using bilinear interpolation operation and downsampled with a 3×3 convolutional operation with a stride of 2, and padding of 1 (*Conv*_3_). The input preparation process for each level of the fusion module is demonstrated in algorithm 1.

**Algorithm 1:** The input preparation process for level *l* of the fusion module

**Input:** The attention module feature outputs *F*′ from all levels

**Output:** The feature maps with the same spatial and channel dimensions *F*

**Parameter:** *n* is the number of the levels of the feature pyramids, *S*_i_ is the spatial dimension of the feature map at level *i*.

**for** *i* = 1,2,..,n **do**

 % convert feature map channel dimension

 *F*′_i_ = *Conv*(*F*′_i_)

 % convert feature map spatial dimension

 **if** *S*_*i*_ = *S*_*l*_ **then**

  *F*_*i*_ = *F*′_i_

 **else if** *S*_*i*_ < *S*_*l*_ **then**

  *F*_i_ = *Bilinear_interpolation*(*F*′_i_)

 **else if** *S*_*i*_ > *S*_*l*_ **then**

  *F*_i_ = *Conv*_3_(*F*′_i_)

**end**

Second, the selection weight is generated by employing global average pooling and global max pooling, followed by a 1×1 convolutional layer with the ReLU activation function and a 1×1 convolutional layer without the softmax activation function as follows:

$$\begin{array}{c} \hat{F}=Conv\left(ReLU\left(Conv\left(GAP\left(\sum_{i=0}^{n}F_{i}\right)+MAP\left(\sum_{i=0}^{n}F_{i}\right)\right)\right)\right), \end{array}$$ (6)

where *F*_*i*_ represents the resized feature maps from each level of the attention module, *n* denotes the number of the level of the feature pyramids, and $\hat{F}$ indicates the selection weight for each feature level. We incorporated the selection weight with the input feature maps using an elementwise operation to select the semantic information from each level of the feature pyramid.

### Auxiliary point detection head

**Fig 5:**
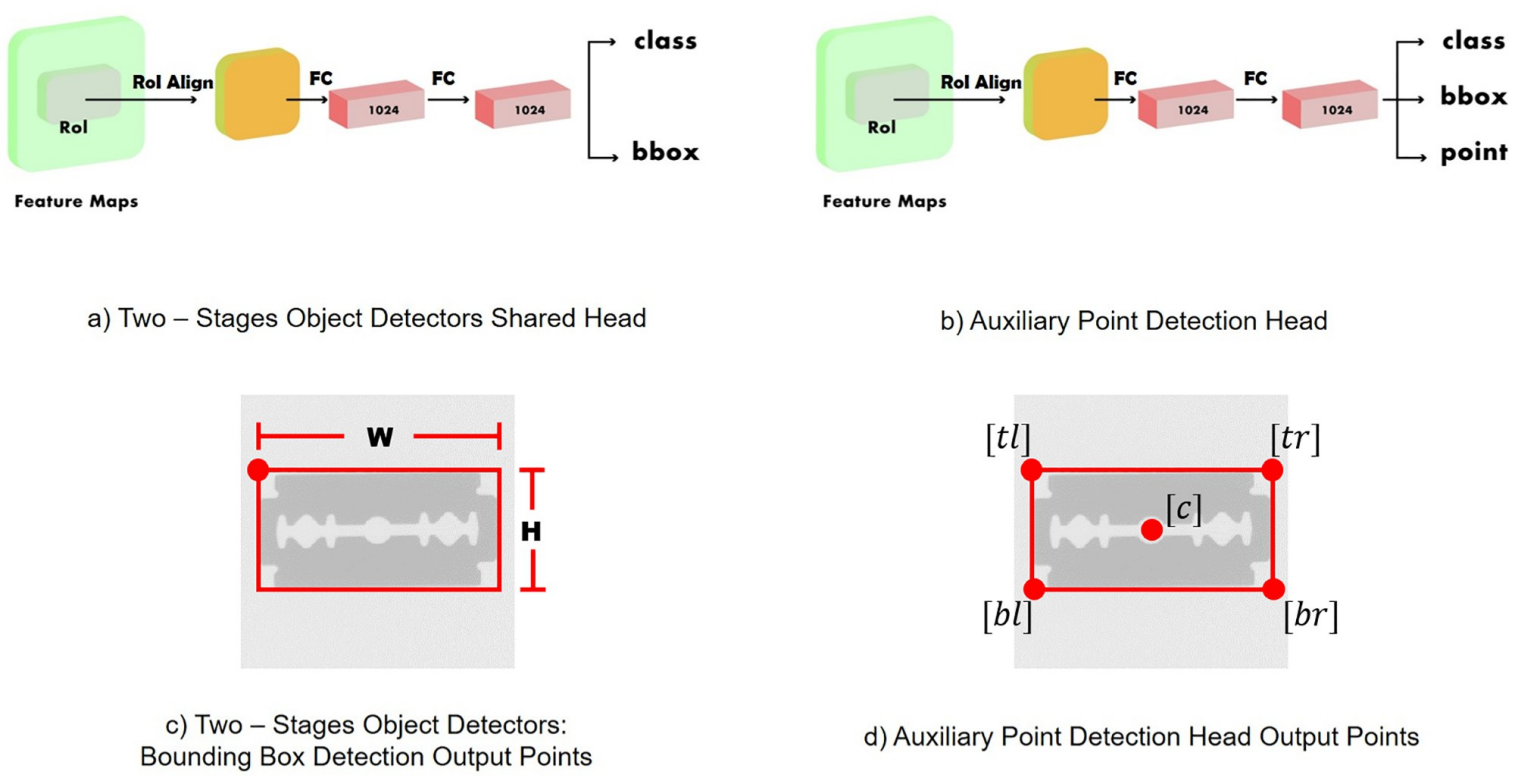
Architectural and bounding box detection-output comparison between the two-stage object detector shared head and proposed auxiliary point detection head: a) two-stage object detector shared head architecture, b) proposed auxiliary point detection architecture, c) two-stage object detectors: bounding box detection-output points, and d) auxiliary point detection head output points.

Several state-of-the-art object detectors have demonstrated outstanding performance in terms of localization by optimizing detection head architecture, such as adding an auxiliary detection head [33, 37]. In this work, we propose the auxiliary point detection head, an additional detection head designed to predict further keypoints of the bounding box to enhance the localization performance of the detectors without additional ground-truth annotation. The point head enhances the localizability of the detector by allowing the detector to learn more information about the object bounding box. In other words, with more information to learn, the detectors indicate the object bounding box more precisely than the baseline detector without the proposed point head. The architecture of the auxiliary point detection head (point head) is illustrated in Fig 5b).

Generally, two-stage object detectors such as Faster R-CNN consist of two prediction heads (Fig 5a)) and predict two outputs for each candidate object, including an object class and the bounding box position defined by four points (*i.e.*, the width, height, and top-left corner *x-* and *y-*coordinates), as listed in Fig 5c). The point head is adopted in the second stage of the object detectors in parallel to the class and bounding box offset prediction head, as depicted in Fig 5b) to predict five points of the bounding box (*i.e.*, top-left corner, top-right corner, bottom-left corner, bottom-right corner, and center of the bounding box) in Fig 5d). The ground truths for additional points were calculated from the ground truths of the bounding box as follows:

$$\begin{array}{c} C_{x}=tl_{x}+\left(\frac{W}{2}\right),\\ C_{y}=tl_{y}+\left(\frac{H}{2}\right),\\ tr_{x}=tl_{x}+W,\\ tr_{y}=tl_{y},\\ bl_{x}=tl_{x},\\ bl_{y}=tl_{y}+H,\\ br_{x}=tl_{x}+W,\\ br_{y}=tl_{y}+H, \end{array}$$

where *C*_*x*_ and *C*_*y*_ denote the *x-* and *y-* coordinates of the center point, *tl*_*x*_ and *tl*_*y*_ are the *x-* and *y-*coordinates of the top-left corner, *tr*_*x*_ and *tr*_*y*_ indicate the *x-* and *y-*coordinates of the top-right corner, *bl*_*x*_ and *bl*_*y*_ represent the *x-* and *y-*coordinates of the bottom-left corner, and *br*_*x*_ and *br*_*y*_ denote the *x-* and *y-*coordinates of the bottom-right corner, respectively. In addition, *H* is the height of the bounding box, and *W* is the width of the bounding box. Thus, 10 output parameters exist for the point head module. Furthermore, the computation cost for this module is minimal compared to the Mask R-CNN because the point head module predicts only 10 output parameters for five bounding box keypoints instead of predicting the segmentation mask in a pixel-to-pixel manner.

## Experiments

### Datasets

The experiment in this study was conducted on two X-ray security inspection datasets belonging to two different domains. The public dataset SIXray [2] contains baggage scanning images. The second dataset is the synthetic cargo scanning dataset CargoX. We designed the experiments to evaluate the performance of the proposed MFA-net on the SIXray and CargoX because we expected the MFA-net to achieve high performance in both image domains, overcome the difficulties of each dataset, and increase the compatibility of the detectors in real-world scenarios.

### SIXray

The SIXray dataset [2] is a set of large-scale security inspection X-ray images with six classes of prohibited items (*i.e.*, guns, knives, wrenches, pliers, scissors, and hammers). The SIXray dataset comprises three subdatasets: SIXray10, SIXray100, and SIXray1000. In this study, we evaluated the proposed object detectors only on SIXray10, in which all 8,929 positive images and exactly 10 times the negative images are included. The dataset was optimized on 80% of the data for training and evaluated on the remaining 20% of the data for testing.

### CargoX

**Fig 6:**
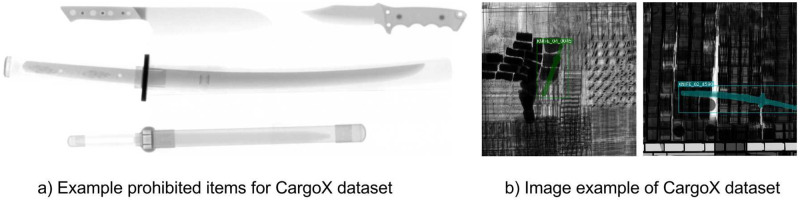
Prohibited items and X-ray security images in the CargoX dataset: a) examples of prohibited items for the CargoX dataset and b) image examples for the CargoX dataset.

The CargoX dataset is a synthetic set of cargo security X-ray images and is the first cargo domain dataset (which we proposed). We generated the cargo X-ray images by cropping them from high-resolution images of vehicles with cargo. It is impossible to accurately label the prohibited items using only the cropped cargo images; thus, we inserted the prohibited items into cropped cargo images. The prohibited items in the CargoX dataset include four different types of knives, which are common weapons for general security screening systems, as presented in Fig 6a).

Inserting items are generated by rotating each type of knife about the *x*-axis, taking X-ray projection images every 10°, and rotating about *y*-axis and taking X-ray images every 45°. We define the images categories by the knife type and roll angle (the rotation angle around *x*-axis) when taking X-ray projection images. First, the knife images are divided into four categories by type. Second, the knife images taken with different roll angle of each group are divided into four groups (*i.e.*, the knife images taken at 45° to 135°, 135° to 225°, 225° to 315°, and 315° to 45° roll angle). Then, we combined the four groups into two groups for each type of knife with a similar shape, combining knife images with roll angle from 45° to 135° and 225° to 315°, and combining knife images with roll angle from 135° to 225° and 315° to 45°. Therefore, the CargoX dataset contains eight prohibited item categories with four knife types, and each type is associated with two categories with different roll angle ranges. We randomly selected a knife image from each category, and inserted it into a random position in the cropped cargo images to generate images for each category.

Moreover, Fig 6b) illustrates the synthetic X-ray images in the CargoX dataset. The synthetic cargo X-ray dataset, CargoX, contains 64,000 X-ray images, consisting of 40,000 images for training, 12,000 images for validation, and 12,000 images for testing. The CargoX dataset has several properties that differ from other security X-ray datasets. First, prohibited items appear with varied scales and viewpoints. Furthermore, the dataset provides the necessary information, such as item categories and bounding box position, and additional information to learn object detection more precisely, which is the object instance segmentation. Finally, the images in this dataset have complex content and noise, as presented in Fig 6b).

### Performance measure

To evaluate the performance of the object detectors, we used the mAP. The mAP is a metric from the *MS COCO* [38] object detection challenge, with three different IoU thresholds. The thresholds include the mAP (mAP scores of 10 IoU thresholds from 0.5 to 0.95, with a step of 0.05), mAP_50_ (mAP scores with an IoU of 0.5), and mAP_75_ (mAP scores with an IoU of 0.75).

### Implementation details

In the implementation, we adopted two-stage object detectors, such as the Faster R-CNN [32], Cascade R-CNN, and Cascade Mask R-CNN [33] with ResNet-50, ResNet-101 [34], and ResNeXt-101 [36] along with the proposed components. The Cascade Mask R-CNN is not implemented for SIXray because the dataset did not provide a mask annotation. Furthermore, we replaced all 3×3 convolutional backbone operations with the multiscale dilated module (MDconv). FusionFPN was employed as the feature fusion network instead of the FPN [27]. The point head was adopted for the detection head. We implemented this model with MMDetection [39], an open-source object detection toolbox based on PyTorch. The models were trained for 20 epochs in the experiment with initial learning rates of 0.005 and 0.02 for SIXray and CargoX, respectively. The learning rate was multiplied by 0.1 after 16 and 19 epochs. We used an SGD optimizer with a batch size of four images per graphics processing unit (GPU) in an environment equipped with NVIDIA Titan Xp GPU, CUDA version 10.2, and PyTorch 1.5.

## Results and discussion

### Ablation study

We perform several ablations to optimize and evaluate the performance of each of the three proposed improvements (*i.e.*, the MDConv module, FusionFPN, and point head).

#### Performance of the multiscale dilated convolutional module

We performed an experiment to optimize the hyperparameter *k*, which is the number of 3× 3 convolutional operations in MDConv. Table 1 reveals that using three 3×3 convolutional operations in MDConv achieved the best mAP in both the SIXray10 and CargoX datasets.

**Table 1:** Effect of the number of parallel 3 × 3 convolutional operations (*k*) in MDConv.

Method	Backbone	*k*	mAP		mAP_50_		mAP_75_	
			SIXray10	CargoX	SIXray10	CargoX	SIXray10	CargoX
Cascade R-CNN [33]	ResNeXt-101 w FPN	1	52.1	70.1	78.4	95.9	59.9	81.1
	ResNeXt-101 w FPN	2	54.9	71.9	78.5	96.5	64.1	81.7
	ResNeXt-101 w FPN	3	**55.4**	**72.5**	**79.6**	**96.5**	**63.2**	**82.2**
	ResNeXt-101 w FPN	4	54.9	68.3	79.3	95.5	62.4	77.6

The effectiveness of the MDconv module was examined on the SIXray10 and CargoX datasets by incorporating MDconv with Cascade R-CNN with FPN. We evaluated the performance of MDConv by comparing the performance with the *MS COCO* state-of-the-art module that optimizes the two-stage object detector backbones with conditional convolution, including switchable atrous convolution (SAC) from DetectoRS [25], the ResNeSt [24] backbone, and deformable convolution [26]. These previous backbone optimization methods, including the MDConv module, were adopted in the ResNeXt-101 baseline backbone. Table 2 indicates that MDConv enhances the mAP values by 2.9% and 2.3% on the SIXray10 and CargoX datasets, respectively. Furthermore, MDConv outperforms other state-of-the-art backbones with 55.4% mAP on SIXray10 and 72.5% mAP on CargoX.

**Table 2:** Comparison of the performance of the MDConv module with previous applications of conditional convolutional modules on the backbones of object detectors on the SIXray10 and CargoX datasets.

Method	Backbone	Addition Module	mAP		mAP_50_		mAP_75_	
			SIXray10	CargoX	SIXray10	CargoX	SIXray10	CargoX
Cascade R-CNN [33]	ResNeXt-101 w FPN	-	52.5	70.2	78.3	95.9	60.6	81.1
	ResNeXt-101 w FPN	SAC [25]	43.0	63.9	67.9	92.8	47.0	71.3
	ResNeSt (s101) [24]	-	51.4	67.1	75.4	94.1	58.6	74.8
	ResNeXt-101 w FPN	DCN [26]	54.2	72.1	78.8	96.4	62.8	**82.4**
	ResNeXt-101 w FPN	MDconv	**55.4**	**72.5**	**79.6**	**96.5**	**63.2**	82.2

#### Performance of the fusion feature pyramid network

To demonstrate the effectiveness of FusionFPN, we substituted the proposed FusionFPN in the Cascade R-CNN with *MS COCO* state-of-the-art feature fusion networks (*i.e.*, FPN [27], PAFPN [28], AugFPN [30], and Libra R-CNN [29]) that were proposed to improve multiscale feature learning. Table 3 indicates that the FusionFPN outperforms other state-of-the-art feature fusion networks with a 54.6% mAP on SIXray10 and 71.3% on CargoX.

**Table 3:** Comparison of the performance of the fusion feature pyramid network with state-of-the-art feature fusion networks on the CargoX dataset.

Method	Backbone	Feature Fusion Network	mAP		mAP_50_		mAP_75_	
			SIXray10	CargoX	SIXray10	CargoX	SIXray10	CargoX
Cascade R-CNN [33]	ResNeXt-101	FPN [27]	52.5	70.7	78.3	96.1	60.6	80.2
	ResNeXt-101	PAFPN [28]	54.2	71.1	79.1	96.1	**63.1**	**81.6**
	ResNeXt-101	AugFPN [30]	53.7	71.1	78.9	96.2	62.1	80.5
	ResNeXt-101	Libra R-CNN	52.7	71.0	77.2	96.4	59.9	81.4
	ResNeXt-101	FusionFPN	**54.6**	**71.3**	**79.3**	**96.5**	62.5	81.3

#### Performance of the auxiliary point detection head

We performed an experiment involving variant predicted point formulations to optimize the number of keypoints outputs of the point head. The experiment on one point uses the supervision of the center of the ground-truth box (*C*). In the two-points case, we used the top-right (*tr*) and the bottom-left (*bl*) points of the ground-truth box. We added the center points to the two-point case in the three-point case. Supervision of the bottom-right (*br*) points is added to the three-point case in the four-point case. The last case is the five-point case, which uses supervision of the top left (*tl*), top right, center, bottom left, and bottom right of the ground-truth box as predicted points of the point head. The experiments in Table 4 deployed Faster R-CNN and Cascade R-CNN with ResNet-50 with FPN and ResNet-101 with FPN. As the number of points increases, the detection accuracy also increases. Adding a point head in the five-points case on the Faster R-CNN with ResNet50 backbone with FPN and ResNet-101 with FPN increases the mAP by 11.2% and 3.8% for SIXray10 and 1.3% and 1.2% for CargoX, respectively. Furthermore, adopting a point head with Cascade R-CNN with a ResNet50 backbone with FPN improves the mAP over the baseline by 3.3% on SIXray10 and 0.3% on CargoX. We also compared the performance of the proposed point head with the keypoint-based prediction object detector. However, CentripetalNet requires the instance segmentation ground truth for learning. Therefore, we only compared the test results on CargoX for a fair comparison. Table 5 indicates that the Cascade R-CNN with the point head outperforms the previous keypoint-based prediction research and achieves a 71.5% mAP.

**Table 4:** Effect of the position of the point in the point head Module.

Method	Backbone	Point Position	mAP		mAP_50_		mAP_75_	
			SIXray10	CargoX	SIXray10	CargoX	SIXray10	CargoX
Faster R-CNN [32]	ResNet-50 w FPN	-	39.5	66.7	72.1	95.9	38.2	75.7
		1[*C*]	45.4	66.7	77.5	95.9	47.8	75.8
		2[*tr*,*bl*]	41.3	64.8	73.2	95.3	45.2	73.2
		3[*tr*,*C*,*bl*]	49.1	67.6	78.4	95.9	55.2	77.4
		4[*tr*,*C*,*bl*,*br*]	49.2	67.8	78.6	95.8	54.6	77.6
		5[*tl*,*tr*,*C*,*bl*,*br*]	**50.7**	**68.0**	**79.3**	**95.8**	**56.7**	**77.9**
Faster R-CNN [32]	ResNet-101 w FPN	-	48.5	67.0	79.0	95.8	54.0	76.3
		1[*C*]	49.8	67.0	78.9	95.9	53.9	76.2
		2[*tr*,*bl*]	44.6	66.5	75.2	95.6	48.4	75.9
		3[*tr*,*C*,*bl*]	51.9	68.1	78.7	96.0	59.1	78.0
		4[*tr*,*C*,*bl*,*br*]	51.8	67.8	79.0	95.6	59.0	78.0
		5[*tl*,*tr*,*C*,*bl*,*br*]	**52.3**	**68.2**	**78.6**	**95.9**	**60.7**	**78.5**
Cascade R-CNN [33]	ResNet-50 w FPN	-	49.7	69.5	78.4	**96.0**	55.3	79.3
		1[*C*]	48.5	67.9	79.5	**96.0**	53.1	78.1
		2[*tr*,*bl*]	40.8	63.4	72.5	93.9	40.4	72.0
		3[*tr*,*C*,*bl*]	51.4	69.3	79.5	95.9	58.4	79.6
		4[*tr*,*C*,*bl*]	52.0	69.4	79.3	95.9	59.4	79.9
		5[*tr*,*C*,*bl*,*br*]	**53.0**	**69.8**	**79.7**	**96.0**	**60.1**	**80.6**

**Table 5:** Comparison of the performance of the point head with state-of-the-art keypoint-based object detectors on the CargoX dataset.

Method	Backbone	mAP	mAP_50_	mAP_75_
CornerNet [40]	Hourglass104	63.7	89.3	72.5
CenterNet [41]	ResNet-101	70.7	**96.2**	79.3
Centripetalnet [42]	Hourglass104	62.8	90.4	70.5
Grid R-CNN [43]	ResNeXt-101 + FPN	70.2	**96.2**	79.3
Cascade R-CNN w Point Head	ResNeXt-101 + FPN	**71.5**	96.0	**82.3**

### Effect of the proposed modules on quantitative performance

This subsection demonstrates the individual effects of the proposed modules (*i.e.,* MDConv, Fusion FPN, and point head) on the MFA-net. We experimented by adopting each module on the Cascade R-CNN with a ResNeXt-101 backbone with the FPN. Table 6 indicates that incorporating the MDConv module into ResNeXt-101 on the Cascade R-CNN improves the mAP values by 2.9% and 2.3% on SIXray10 and CargoX, respectively. Furthermore, replacing the FPN with the proposed FusionFPN achieves a 54.6% mAP on SIXray10, an increase of 2.1% over the baseline, and a 71.3% mAP on CargoX, an increase of 1.1% over the baseline. In addition, adopting the point head leads to an mAP increase of 3.5% and 1.3% on SIXray10 and CargoX, respectively. Furthermore, incorporating all contributions with the Cascade R-CNN with a ResNeXt-101 backbone with the FPN increases the mAP values by 5.5% and 2.5% on SIXray and CargoX, respectively.

**Table 6:** Effect of each proposed module on the SIXray10 and CargoX datasets.

Method	Backbone	MDConv	Fusion FPN	Point Head	mAP		mAP_50_		mAP_75_	
					SIXray10	CargoX	SIXray10	CargoX	SIXray10	CargoX
Cascade R-CNN [33]	ResNeXt-101 w FPN	✘	✘	✘	52.5	70.2	78.3	95.9	60.6	81.1
		✔	✘	✘	55.4	72.5	79.6	**96.5**	63.2	82.2
		✘	✔	✘	54.6	71.3	79.3	**96.5**	62.5	81.3
		✘	✘	✔	56.0	71.5	80.0	96.0	65.5	82.3
		✔	✔	✔	**58.0**	**72.7**	**80.3**	**96.5**	**68.3**	**83.5**

### Effect of the proposed modules on qualitative performance

**Fig 7:**
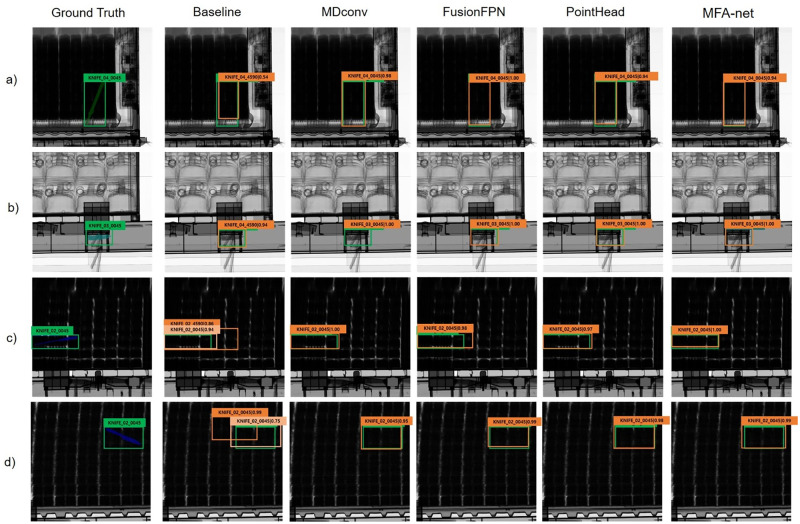
Visualization of the effect of the proposed MDConv, FusionFPN, point head, and MFA-net with all the proposed modules on CargoX.

**Fig 8:**
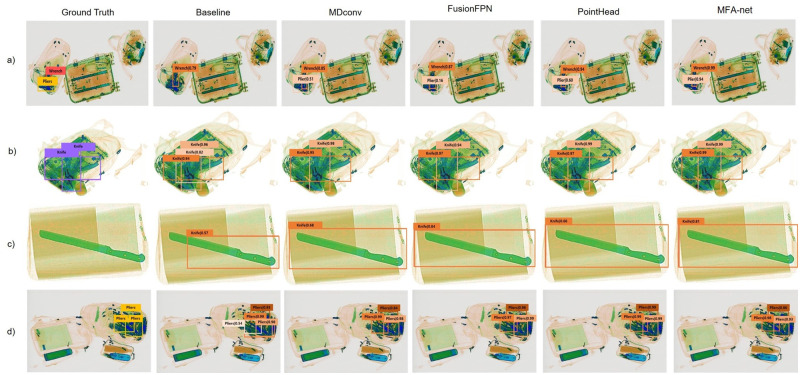
Visualization of the effect of the proposed MDConv, FusionFPN, point head, and MFA-net with all the proposed modules on SIXray [2].

For a quality performance analysis of the proposed modules, we visualized the effect of each proposed component on CargoX and SIXray. We incorporated each proposed component into the baseline (*i.e.,* the Cascade R-CNN with a ResNeXt-101 backbone with the FPN) and compared the detection performance with the baseline. Fig 7a) indicates that each of the three proposed modules enhances the localization performance to predict the position of an object’s bounding box more accurately. The proposed components also refine the classification performance, as detailed in Fig 7b). Furthermore, Fig 8a) indicates that adopting the proposed modules can decrease false-negative prediction cases of the baseline detectors. Besides decreasing false-negative prediction cases compared to the baseline, Fig 7c) and 7d) and Fig 8b) and 8d) reveal that the proposed modules can also decrease the number of false-positive prediction cases. Fig 8c) illustrates that the proposed modules can detect prohibited objects and provide better performance in terms of localization in false-negative ground-truth cases.

### Comparison with state-of-the-art detectors

Previous studies [2, 44] on SIXray usually evaluated the object detector performance by determining the accuracy in the mAP of the classification and localization, which is different from the general object detection task on natural images (*e.g.*, *MS COCO* and *PASCAL VOC*). However, we evaluated the performance of MFA-net using the *MS COCO* mAP metric on the SIXray and CargoX datasets. Therefore, we conducted experiments on SIXray10 and CargoX by comparing the performance of the MFA-net with the *MS COCO* state-of-the-art object detectors. Table 7 compares the MFA-net with the *MS COCO* state-of-the-art one- and two-stage detectors. Compared to the Faster R-CNN with a ResNeXt-101 backbone with the FPN, the MFA-net-based Faster R-CNN increases mAP values by 3.3% and 2.5% on SIXray10 and CargoX, respectively. Furthermore, the MFA-net-based Cascade R-CNN with ResNeXt-101 improves the mAP values over the baseline by 5.5% on SIXray10 and 2.5% on CargoX. Additionally, the MFA-net outperforms the state-of-the-art approaches in SIXray and CargoX and achieves the best performance with mAP values of 58.0% and 75.2%, respectively.

**Table 7:** Performance comparison with state-of-the-art algorithms on the SIXray and CargoX datasets.

Method	Backbone	# Params (M)	mAP		mAP_50_		mAP_75_	
			SIXray10	CargoX	SIXray10	CargoX	SIXray10	CargoX
SSD300+DOAM [3]	VGG16	24.71	45.9	63.3	68.8	93.7	53.0	70.7
SSD300 [45]	VGG16	24.68	45.6	55.9	68.9	89.7	52.1	60.4
SSD512 [45]	VGG16	25.42	48.6	65.5	72.3	94.9	55.8	73.8
YOLOv3 [46]	DarkNet-53	61.56	39.5	56.9	73.8	93.1	37.8	62.3
FCOS [47]	ResNet-101 w FPN	50.89	46.8	66.8	76.7	95.5	51.0	76.8
RetiNaNet [48]	ResNext-101 w FPN	54.88	40.5	66.7	71.5	95.3	41.1	76.6
CornerNet [40]	Hourglass104	200.95	-	63.7	-	89.3	-	72.5
Grid R-CNN [43]	ResNext-101 w FPN	82.88	-	70.2	-	96.2	-	79.3
Libra R-CNN (Faster R-CNN) [29]	ResNeXt-101	87.84	48.9	68.1	76.7	96.4	56.3	77.5
HRNet (Faster R-CNN) [49]	HRNetV2p-40	63.16	50.3	66.4	78.5	95.9	57.3	74.7
HRNet (Cascade R-CNN) [49]	HRNetV2p-40	90.95	55.1	71.5	78.9	96.5	64.3	80.6
ResNeSt [24]	S-101 w FPN	91.52	51.4	67.1	75.4	94.1	58.6	74.8
Faster R-CNN [32]	ResNet-50 w FPN	41.16	39.5	66.7	72.1	95.9	38.2	75.7
MFA-Net (Faster R-CNN)	ResNet-50	56.72	51.2	68.3	78.8	96.5	57.7	78.1
Faster R-CNN [32]	ResNet-101 w FPN	60.15	48.5	67.0	79.0	95.8	54.0	76.3
MFA-Net(Faster R-CNN)	ResNet-101	86.87	54.0	69.2	79.7	96.7	61.8	77.8
Faster R-CNN [32]	ResNeXt-101 w FPN	59.79	49.8	68.5	78.7	96.2	56.4	79.0
MFA-Net (Faster R-CNN)	ResNeXt-101	75.04	53.1	71.0	77.9	96.9	61.3	81.4
Cascade R-CNN [33]	ResNet-50 w FPN	68.95	49.0	69.5	78.4	96.0	55.2	79.3
MFA-Net (Cascade R-CNN)	ResNet-50	84.46	55.4	71.1	79.6	96.4	64.5	81.1
Cascade R-CNN [33]	ResNet-101 w FPN	87.94	53.0	70.1	79.4	95.9	61.2	80.2
MFA-Net (Cascade R-CNN)	ResNet-101	114.61	57.5	71.3	80.8	96.3	66.8	81.4
Cascade R-CNN [33]	ResNeXt-101 w FPN	87.58	52.5	70.2	78.3	95.9	60.6	81.1
MFA-Net (Cascade R-CNN)	ResNeXt-101	102.78	**58.0**	72.7	**80.3**	96.5	**68.3**	83.5
Cascade mask R-CNN [33]	ResNet-50 w FPN	76.82	-	71.9	-	95.5	-	89.4
MFA-Net (Cascade mask R-CNN)	ResNet-50	92.33	-	72.2	-	96.1	-	89.5
Cascade mask R-CNN [33]	ResNet-101 w FPN	95.81	-	72.1	-	95.5	-	89.4
MFA-Net (Cascade mask R-CNN)	ResNet-101	122.48	-	72.4	-	95.9	-	89.9
Cascade mask R-CNN [33]	ResNeXt-101 w FPN	98.45	-	72.6	-	96.7	-	82.3
MFA-Net (Cascade mask R-CNN)	ResNeXt-101	110.65	-	**75.2**	-	**96.7**	-	**84.5**

In term of the computational efficiency, we compared the proposed method with state-of-the-art object detectors. Comparing the performance of the proposed MFA-net with other object detection methods, the MFA-net outperform other methods in term of detection accuracy (*e.g.*, classification accuracy and object bounding box localization accuracy). However, as demonstrated in Table 7, the proposed MFA-net has more trainable parameters than the baseline models. Specifically, the MFA-Net (Faster R-CNN) with the ResNet-50 backbone requires about 15.5 million more parameters (120.67 ms more inference time) than the Faster R-CNN baseline methods to improve the accuracy by 1.6% mAP for the CargoX dataset because the MFA-net consists of three plug-and-play modules incorporated into the baseline models. Accuracy is of the utmost importance for security checks, and it takes seconds to minutes for a human to inspect a cargo image; thus, the accompanying increase in estimated time is negligible. In addition, the inspection system generally has high-specification computing power; therefore, it is expected that the increase in the estimation time due to the accompanying parameter increase can be further minimized.

## Conclusions

In this study, we investigated the feasibility of object detection of contraband items in X-ray security images, an essential application in industry to maintain transport security against smuggled items in various forms. However, this promising application has not yet been studied computer vision because few well-established X-ray datasets are available. To promote research in this field, we constructed a cargo X-ray security image dataset named CargoX that provides realistic content with complex content and various geometrics and evaluated object detection algorithms on the cargo domain dataset for the first time.

Moreover, we proposed the MFA-net, an object detector, to identify prohibited items and overcome the challenges of X-ray security images over natural images, such as overlapping objects, heavy cluttering, geometric variation, and complex content. The MFA-net consists of three main plug-and-play modules (*i.e.*, MDConv module, FusionFPN, and point head) that can be easily mounted into most two-stage object detection networks. We evaluated the performance of the MFA-net using two X-ray security datasets from the baggage domain SIXray and the cargo domain CargoX. The experiment results reveal that each proposed module outperformed the previous methods with a similar optimization concept. Furthermore, the MFA-net significantly improves the performance of the baseline detectors qualitatively and quantitatively in both domains.

Overall, we demonstrated the proposed detector’s potential application in real-world inspections. Its usage might reduce the need for the intervention of human operators and eliminate human error factors in the inspection process.
